# Metabolic acidosis is associated with pulse wave velocity in chronic kidney disease: Results from the KNOW-CKD Study

**DOI:** 10.1038/s41598-019-52499-6

**Published:** 2019-11-06

**Authors:** Hyo Jin Kim, Eunjeong Kang, Hyunjin Ryu, Miyeun Han, Kyu-Beck Lee, Yong-Soo Kim, Suah Sung, Curie Ahn, Kook-Hwan Oh

**Affiliations:** 1 Department of Internal Medicine, Pusan National University Hospital, Busan, Korea; 2 Department of Internal Medicine, Seoul National University Hospital, Seoul, Korea; 3 Department of Internal Medicine, Seoul National University College of Medicine, Seoul, Korea; 4 Department of Internal Medicine, Kangbuk Samsung Hospital, Sungkyunkwan University School of Medicine, Seoul, Korea; 5 Department of Internal Medicine, Seoul St. Mary’s Hospital, College of Medicine, The Catholic University of Korea, Seoul, Korea; 6 Department of Internal Medicine, Eulji Medical Center, Eulji University, Seoul, Korea

**Keywords:** Kidney, Chronic kidney disease

## Abstract

Metabolic acidosis is common in chronic kidney disease (CKD) and may have various deleterious consequences. Arterial stiffness in CKD patients is associated with poor cardiovascular outcomes. The present study aimed to evaluate the association between serum bicarbonate and arterial stiffness using the baseline cross-sectional data set of a large-scale Korean CKD cohort. 2,238 CKD patients were enrolled in the KoreaN Cohort Study for Outcome in Patients With Chronic Kidney Disease (KNOW-CKD) from 2011 to 2016. The present study was conducted on 1,659 patients included in this cohort with baseline serum bicarbonate and brachial-to-ankle pulse wave velocity (baPWV) data. Metabolic acidosis was defined as a serum bicarbonate level of <22 mmol/L, and baPWV was used as a surrogate of arterial stiffness. Mean serum bicarbonate was 25.8 ± 3.6 mmol/L. 210 (12.7%) patients had metabolic acidosis. baPWV was significantly higher in patients with metabolic acidosis (*P* < 0.001) and showed a significant inverse correlation with serum bicarbonate (Unstandardized β −16.0 cm/sec; 95% CI −20.5, −11.4; *P* < 0.001) in an unadjusted model, which was retained after adjustment (Unstandardized β −5.4 cm/sec; 95% CI −9.9, −1.0; *P* = 0.017). Metabolic acidosis was found to be associated with a high baPWV in pre-dialysis CKD patients.

## Introduction

The kidney plays a major role in the maintenance of systemic acid-base balance, but the ability of the kidney to excrete ammonium or reabsorb bicarbonate in response to daily acid load is impaired in chronic kidney disease (CKD)^1–3^. Therefore, metabolic acidosis, usually indicated by a low serum bicarbonate level, is common in CKD. The prevalence of metabolic acidosis depends on the definition used, for example, when metabolic acidosis was defined as a serum bicarbonate concentration of <22 mmol/L, 2.3 to 13% of patients with CKD stage 3 and 19 to 37% of patients with CKD stage 4 exhibited metabolic acidosis^4,5^. Notably, metabolic acidosis in CKD is associated with chronic inflammation, muscle protein degradation with muscle wasting, bone disease, impaired glucose tolerance, impaired albumin synthesis, accelerated CKD progression, and heart disease^2,6^.

Arterial stiffness is related to medial calcification in muscular and elastic arteries and is used as a surrogate marker of arteriosclerosis^7^. Arterial stiffness is common in CKD and a risk factor of poor cardiovascular outcomes in CKD^8^. Furthermore, in CKD patients, a number of factors such as age, physical inactivity, smoking, hypertension, hyperglycemia, hyperphosphatemia, and chronic inflammation can accelerate vascular calcification, which is a major mediator of arterial stiffness^7^. Pulse wave velocity (PWV) provides a standard means of evaluating vascular stiffness^9,10^, and carotid-femoral PWV (cfPWV; a measure of aortic stiffness) and brachial-ankle PWV (baPWV; a measure of central and peripheral arterial stiffness) are the most frequently used measures of arterial stiffness. baPWV and cfPWV are well correlated and both exhibit similar associations with the risk factors of cardiovascular disease^11,12^.

In a previous study, PWV changes after 1-year of hemodialysis were related to serum bicarbonate levels in hemodialysis patients, that is, the lowest total carbon dioxide (TCO_2_) group (TCO_2_ <20 mmol/L) presented significantly greater PWV increases over the 1-year study period^13^. In another study, serum bicarbonate levels inversely associated with cfPWV in hemodialysis patients (r = −0.719, *P* = 0.001)^14^. Metabolic acidosis activates the buffer system, which results in serum calcium and phosphate ion increases due to bone resorption^15,16^. In addition, inflammation and insulin resistance, which are the adverse consequences of metabolic acidosis, may cause arteriosclerosis^17^. These observations suggest metabolic acidosis and arterial stiffness may be closely related, but few have investigated the link between metabolic acidosis and arterial stiffness in CKD patients. Accordingly, the present study was undertaken to investigate the association between metabolic acidosis and arterial stiffness using serum bicarbonate levels and baPWV as surrogates, respectively, using the baseline data set of a large-scale Korean CKD cohort.

## Results

### Demographic and baseline clinical characteristics of the study subjects

The clinical characteristics of the 1,659 study subjects at enrollment are presented in Table 1. Mean age was 53.4 ± 12.4 years, and 1,020 (61.5%) were male. Mean estimated glomerular filtration rate (eGFR) was 53.3 ± 31.1 mL/min/1.73 m^2^. Patients with diabetes mellitus (DM) or hypertension (HTN) comprised 34.7% and 95.7% of the study subjects, respectively. Mean serum bicarbonate level was 25.8 ± 3.6 mmol/L. Subjects in the low serum bicarbonate group had higher prevalence of DM (*P* = 0.015), HTN (*P* = 0.031), and preexisting cardiovascular disease (CVD) (*P* < 0.001) than subjects in the other three groups. Furthermore, in the low serum bicarbonate group, eGFR (*P* < 0.001) was lower, proteinuria (*P* < 0.001) was higher, serum albumin (*P* < 0.001) and total cholesterol (*P* < 0.001) levels were lower, and serum phosphorous (*P* < 0.001), and intact parathyroid hormone (iPTH) (*P* < 0.001) levels were higher than in the other three groups. Dietary protein intake (DPI) was lower in the low and lower normal bicarbonate groups than in the other two groups (*P* = 0.002), but was higher than the Kidney Disease Improving Global Outcomes (KDIGO) guideline recommended dose for CKD patients (<0.8 g/kg/day) for all TCO_2_ groups. Furthermore, members of the low serum bicarbonate group were being administered more diuretics than members of the other groups (*P* < 0.001).

**Table 1:** Clinical characteristics of the study subjects at enrollment stratified by serum bicarbonate concentration.

Characteristics	Total (N = 1,659)	Serum TCO_2_				*P*-value	*P* for trend
		Low (<22 mmol/L) (n = 210)	Lower normal (22–26 mmol/L) (n = 697)	Higher normal (26.1–29.9 mmol/L) (n = 524)	High (≥30 mmol/L) (n = 228)		
Total CO_2_	25.8 ± 3.6	19.6 ± 1.9	24.2 ± 1.3	27.9 ± 0.9	31.3 ± 1.4	<0.001	<0.001
Age (mean ± SD)	53.4 ± 12.4	54.2 ± 12.0	54.1 ± 12.5	52.5 ± 12.3	52.5 ± 12.5	0.069	0.063
Sex, male, n (%)	1,020 (61.5)	125 (59.5)	428 (61.4)	317 (60.5)	150 (65.8)	0.504	0.288
BMI (kg/m^2^)	24.6 ± 3.5	24.0 ± 3.4	24.7 ± 3.4	24.6 ± 3.6	24.7 ± 3.2	0.067	0.048
SBP (mmHg)	127.5 ± 15.3	129.7 ± 18.0	127.1 ± 15.3	127.3 ± 14.8	127.4 ± 13.5	0.194	0.146
DBP (mmHg)	77.0 ± 10.9	77.0 ± 11.6	76.3 ± 10.7	77.8 ± 11.1	77.2 ± 10.2	0.145	0.519
MAP (mmHg)	93.8 ± 11.2	94.5 ± 12.3	93.3 ± 11.0	94.3 ± 11.3	93.9 ± 10.4	0.331	0.809
Heart rate (/min)	73.4 ± 12.9	73.4 ± 13.5	72.7 ± 12.6	74.5 ± 13.1	73.4 ± 12.7	0.121	0.595
DM, n (%)	574 (34.7)	84 (40.0)	259 (37.2)	168 (32.1)	63 (28.0)	0.015	0.001
HTN, n (%)	1586 (95.7)	206 (98.1)	672 (96.6)	495 (94.5)	213 (93.4)	0.031	0.003
Preexisting CV disease, n (%)	279 (16.8)	56 (26.7)	120 (17.2)	77 (14.7)	26 (11.4)	<0.001	<0.001
CAD, n (%)	115 (7.0)	26 (12.4)	52 (7.5)	27 (5.2)	10 (4.4)	0.002	<0.001
Cerebrovascular disease, n (%)	113 (6.8)	17 (8.1)	52 (7.5)	34 (6.5)	10 (4.4)	0.361	0.088
HF, n (%)	28 (1.7)	10 (4.8)	11 (1.6)	6 (1.1)	1 (0.4)	0.002	0.001
Arrhythmia, n (%)	36 (2.2)	10 (4.8)	8 (1.1)	13 (2.5)	5 (2.2)	0.016	0.187
PVD, n (%)	63 (3.8)	12 (5.7)	27 (3.9)	18 (3.4)	6 (2.6)	0.368	0.101
Cause of CKD						<0.001	0.229
DN, n (%)	387 (23.3)	61 (29.0)	193 (27.7)	104 (19.8)	29 (12.7)		
Hypertension, n (%)	288 (17.4)	45 (21.4)	120 (17.2)	87 (16.6)	36 (15.8)		
GN, n (%)	565 (34.1)	67 (31.9)	228 (32.7)	174 (33.2)	96 (42.1)		
PKD, n (%)	311 (18.7)	22 (10.5)	111 (15.9)	126 (24.0)	52 (22.8)		
Others, n (%)	108 (6.5)	15 (7.1)	45 (6.5)	33 (6.3)	15 (6.6)		
Smoking status, n (%)						0.660	0.169
Never	888 (53.5)	109 (51.9)	359 (51.5)	292 (55.7)	128 (56.1)		
Former	514 (31.0)	70 (33.3)	221 (31.7)	153 (29.2)	70 (30.7)		
Current	257 (15.5)	31 (14.8)	117 (16.8)	79 (15.1)	30 (13.2)		
eGFR(mL/min/1.73 m^2^)	53.3 ± 31.1	27.8 ± 19.0	45.5 ± 28.0	65.7 ± 30.0	72.3 ± 27.6	<0.001	<0.001
Hemoglobin (g/dL)	12.8 ± 2.0	11.2 ± 1.8	12.6 ± 2.0	13.4 ± 1.9	13.8 ± 1.8	<0.001	<0.001
Uric acid (mg/dL)	7.0 ± 1.9	7.6 ± 1.9	7.3 ± 2.0	6.6 ± 1.9	6.5 ± 1.7	<0.001	<0.001
Albumin (g/dL)	4.2 ± 0.4	4.0 ± 0.4	4.1 ± 0.5	4.3 ± 0.4	4.3 ± 0.4	<0.001	<0.001
Total cholesterol (mg/dL)	174.6 ± 38.8	164.5 ± 44.3	173.3 ± 38.7	177.9 ± 36.7	180.2 ± 36.7	<0.001	<0.001
CRP, median, (Q1, Q3) (mg/L)	0.6 (0.2, 1.6)	0.7 (0.4, 2.1)	0.6 (0.2, 1.7)	0.5 (0.2, 1.4)	0.5 (0.2, 1.4)	0.001	<0.001
Phosphorus (mg/dL)	3.7 ± 0.7	4.2 ± 0.9	3.7 ± 0.7	3.5 ± 0.6	3.6 ± 0.6	<0.001	<0.001
*Corrected Ca (mg/dL)	9.0 ± 0.4	8.8 ± 0.5	9.0 ± 0.4	9.1 ± 0.4	9.1 ± 0.4	<0.001	<0.001
iPTH, median (Q1, Q3) (pg/mL)	50.9 (33.7, 83.7)	91.6 (53.3, 144.7)	56.2 (36.9, 94.7)	43.0 (30.2, 65.0)	39.1 (27.7, 54.0)	<0.001	<0.001
UPCR (Q1, Q3) (g/g)	0.49 (0.15, 1.48)	1.08 (0.39, 2.25)	0.58 (0.19, 1.83)	0.34 (0.10, 0.93)	0.27 (0.07, 0.81)	<0.001	<0.001
DPI (g/kg/day)	0.95 ± 0.26	0.93 ± 0.28	0.92 ± 0.25	0.98 ± 0.28	0.96 ± 0.25	0.002	0.060
Medications							
ACEi or ARB, n (%)	1418 (85.5)	181 (86.2)	589 (84.5)	448 (85.5)	200 (87.7)	0.673	0.665
Diuretics, n (%)	498 (30.0)	83 (39.5)	220 (31.6)	125 (23.9)	70 (30.7)	<0.001	0.003
^†^Phosphate binder, n (%)	154 (9.3)	31 (14.8)	68 (9.8)	37 (7.1)	18 (7.9)	0.010	0.005

*Corrected Ca (mg/dL) = measured total Ca (mg/dL) + 0.8 × [4 − measured serum albumin (g/dL)].

^†^Only calcium-based phosphate binders were prescribed to members of our patient cohort.

TCO_2_, total CO_2_; SD, standard deviation; BMI, body mass index; SBP, systolic blood pressure; DBP, diastolic blood pressure; MAP, mean arterial pressure; DM, diabetes mellitus; HTN, hypertension; CV, cardiovascular; CAD, coronary artery disease; HF, heart failure; PVD, peripheral vascular disease; CKD, chronic kidney disease; DN, diabetic nephropathy; PKD, polycystic kidney disease; eGFR, estimated glomerular filtration rate as determined by the CKD-EPI creatinine equation; CRP, C-reactive protein; Ca, calcium; iPTH, intact parathyroid hormone; UPCR, urine protein creatinine ratio; DPI, dietary protein intake; ACEi, angiotensin converting enzyme inhibitor; ARB, angiotensin II receptor blocker.

There were similar clinical characteristics between subjects excluded from the study and those included in the study (Supplemental Table 1). Age, mean arterial pressure (MAP), eGFR, and prevalence of DM and HTN were similar between those groups. Prevalence of CVD was higher in study subjects.

### Prevalence of metabolic acidosis and its characteristics

**Figure 1:**
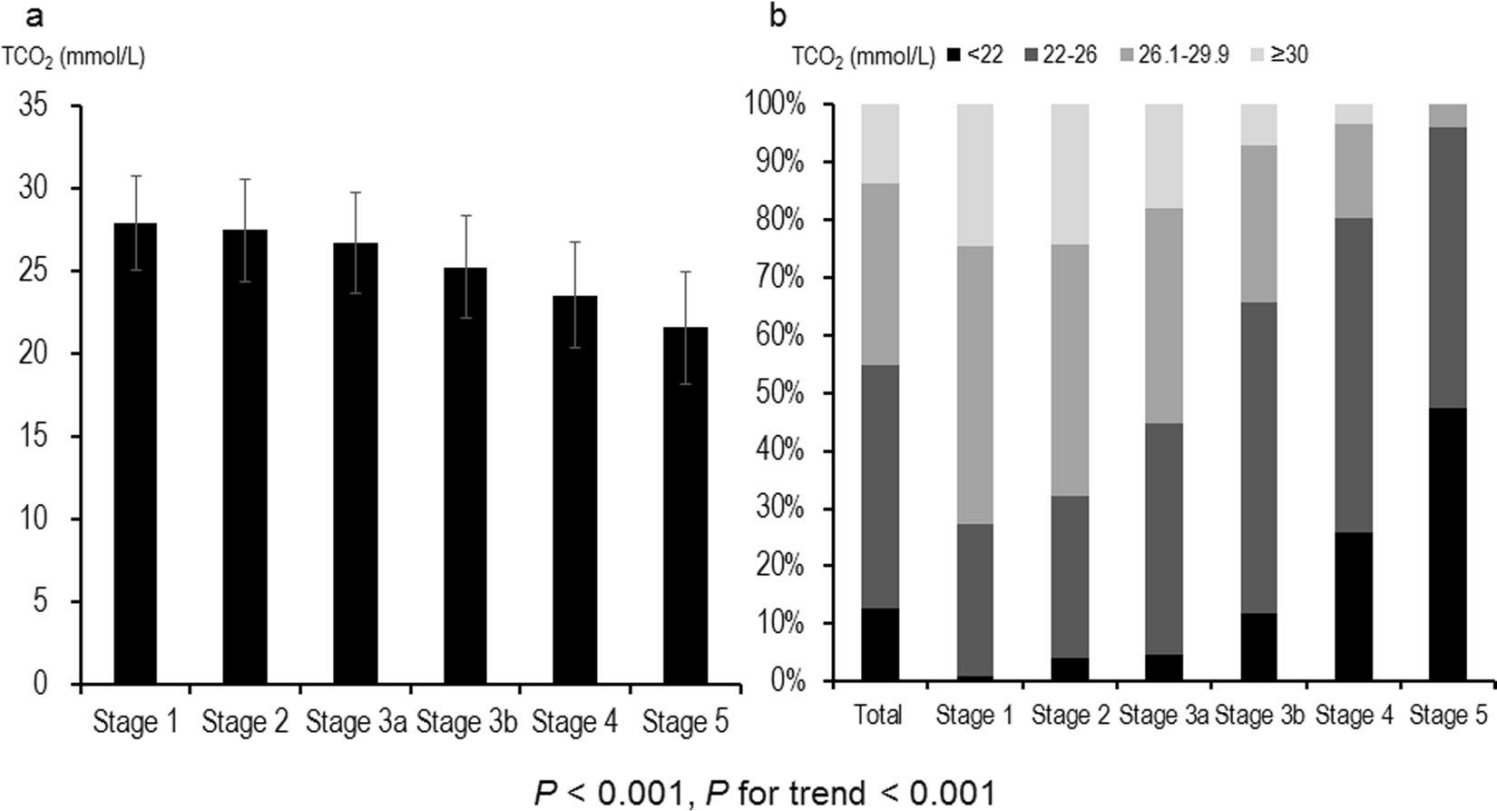
Distributions of serum bicarbonate concentrations and metabolic acidosis prevalences across CKD stages. Advanced CKD stages were associated with a lower serum bicarbonate level (*P* < 0.001, *P* for linear trend <0.001; **a**). Two hundred and ten (12.7%) patients had metabolic acidosis. The prevalence of metabolic acidosis was higher in advanced CKD (*P* < 0.001, *P* for linear trend <0.001; **b**); 26.0% and 47.4% of CKD stage 4 and 5 patients, respectively, exhibited metabolic acidosis. CKD, chronic kidney disease.

**Figure 2:**
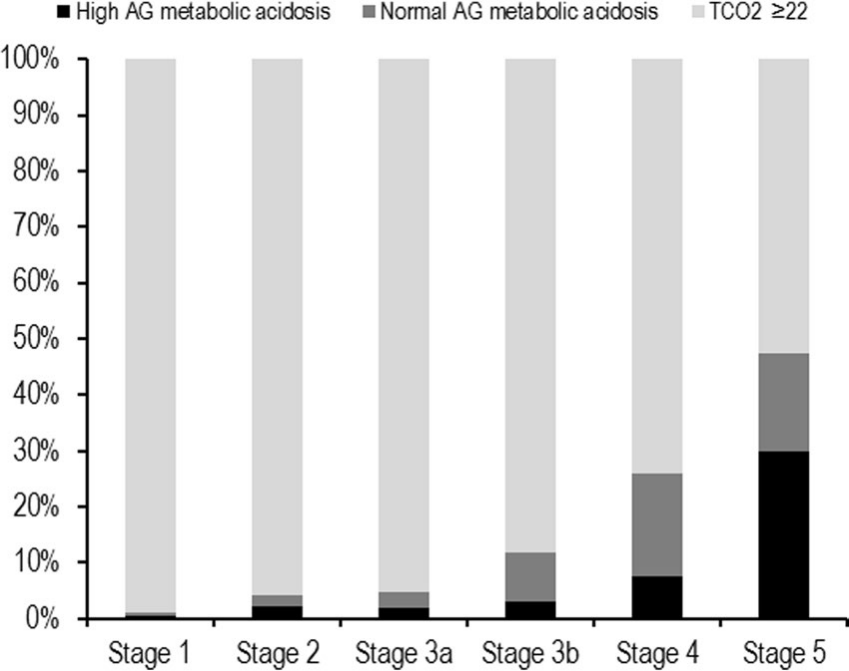
Pattern of metabolic acidosis across CKD stages. High AG metabolic acidosis accounted for 33.3% of total metabolic acidosis in CKD stage 1 and for 63.0% in CKD stage 5. CKD, chronic kidney disease; AG, anionic gap.

Advanced CKD stages were associated with a lower serum bicarbonate level (*P* < 0.001, *P* for linear trend <0.001; Fig. 1a). Two hundred and ten (12.7%) of the study subjects had metabolic acidosis, and its prevalence was greater in advanced CKD (*P* < 0.001, *P* for linear trend <0.001; Fig. 1b); 26.0% and 47.4% of patients with CKD stage 4 or 5 exhibited metabolic acidosis, respectively. When metabolic acidosis was divided into high anionic gap (AG) and normal AG metabolic acidosis, high anionic gap AG metabolic acidosis accounted for 33.3% of total metabolic acidosis in CKD stage 1 and for 63.0% in CKD stage 5 (Fig. 2).

### Pulse wave velocity and the association between serum bicarbonate and arterial stiffness

**Figure 3:**
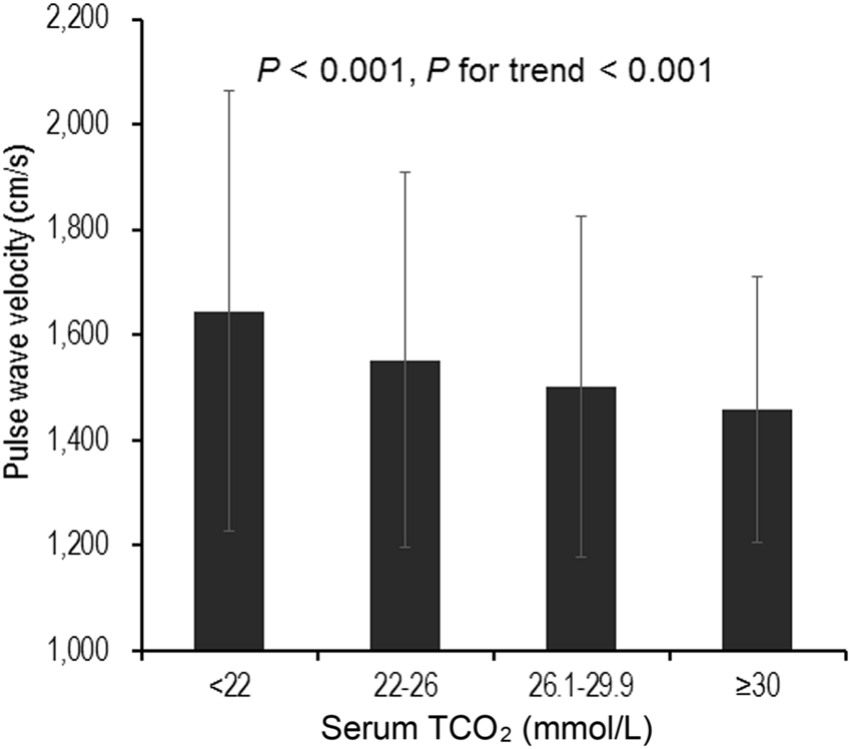
Brachial-to-ankle pulse wave velocity according to the serum bicarbonate concentration. baPWV was significantly higher in low serum bicarbonate group than in the other three groups (*P* < 0.001, *P* for linear trend <0.001). baPWV, brachial-to-ankle pulse wave velocity.

For all study subjects, mean baPWV was 1534 ± 347 cm/sec, and baPWV was significantly higher in low serum bicarbonate group than in the other three groups (*P* < 0.001, *P* for linear trend <0.001; Fig. 3). Table 2 summarizes the results of linear regression analysis of the association between serum bicarbonate and baPWV. baPWV had an inverse association with serum bicarbonate when treated as a continuous variable in the unadjusted model (Unstandardized β −16.0 cm/sec; 95% CI −20.5, −11.4; *P* < 0.001), and this association remained after sequential adjustments for confounders (models 2–4). In the model adjusted for age, sex, HTN, DM, preexisting CVD, systolic blood pressure, body mass index (BMI), eGFR, log proteinuria, hemoglobin, albumin, phosphorus, corrected calcium, total cholesterol, log iPTH, angiotensin converting enzyme inhibitor or angiotensin II receptor blocker use, and diuretics use, baPWV was significantly associated with serum bicarbonate (Unstandardized β −5.4 cm/sec; 95% CI −9.9, −1.0; *P* = 0.017). baPWV showed also a significant inverse association with serum bicarbonate when treated as categorical variables. TCO_2_ < 22 mmol/L was associated with higher baPWV compared to a concentration of ≥30 mmol/L (unstandardized beta = 80.2 cm/sec; 95% CI 20.2, 140.2; *P* = 0.009) in a fully adjusted model (model 4).

**Table 2:** Association between serum bicarbonate concentration and brachial-to-ankle pulse wave velocity.

	Model 1	*P* value	Model 2	*P* value	Model 3	*P* value	Model 4	*P* value
	Unstandardized β (95% CI)		Unstandardized β (95% CI)		Unstandardized β (95% CI)		Unstandardized β (95% CI)	
TCO_2_ (per 1 mmol/L increase)	−16.0 (−20.5, −11.4)	<0.001	−10.3 (−14.0, −6.6)	<0.001	−6.5 (−10.8, −2.2)	0.003	−5.4 (−9.9, −1.0)	0.017
TCO_2_ group								
Low (<22 mmol/L)	187.3 (123.251.6)	<0.001	129.3 (77.2, 181.5)	<0.001	89.2 (31.2, 147.1)	0.003	80.2 (20.2, 140.2)	0.009
Low normal (22–26 mmol/L)	94.2 (42.9, 145.5)	<0.001	61.3 (19.8, 102.8)	0.004	35.1 (−9.0, 79.3)	0.118	35.8 (−9.1, 80.7	0.118
Higher normal (26.1–29.9 mmol/L)	43.6 (−9.7, 96.9)	0.109	30.4 (−12.6, 73.4)	0.166	27.8 (−15.9, 71.5)	0.212	30.5 (−13.8, 74.8)	0.178
High (≥30 mmol/L)	0 (Reference)		0 (Reference)		0 (Reference)		0 (Reference)	

Model 1: Unadjusted.

Model 2: Adjusted for age, sex, HTN, DM, preexisting CVD, mean arterial pressure, heart rate, BMI.

Model 3: Model 2 + adjustment for eGFR, log UPCR.

Model 4: Model 3 + adjustment for hemoglobin, albumin, phosphorus, corrected Ca, total cholesterol, log iPTH, ACEi or ARB use, and diuretics use.

CI, confidence interval; TCO_2_, total CO_2_; DM, diabetes mellitus; HTN, hypertension; CVD, cardiovascular disease; BMI, body mass index; eGFR, estimated glomerular filtration rate as determined by the CKD-EPI creatinine equation; Ca, calcium; iPTH, intact parathyroid hormone; UPCR, urine protein creatinine ratio; ACEi, angiotensin converting enzyme inhibitor; ARB, angiotensin II receptor blocker.

## Discussion

The kidney is principally responsible for regulating acid-base balance, and thus, a decrease in renal function results in metabolic acidosis. During the course of CKD, a positive acid balance decreases serum bicarbonate concentration. In the present study, 12.7% of the 1,659 CKD patients had metabolic acidosis (TCO_2_ < 22 mmol/L) and in stage 4 and 5 CKD, 26.0% and 47.4% of patients, respectively, had metabolic acidosis. Furthermore, baPWV was significantly higher in the low serum bicarbonate group and multivariable analysis after adjustment showed baPWV was significantly and inversely correlated with serum bicarbonate levels.

In a recent study of community-living elders, there was no association of serum bicarbonate concentration or arterialized venous pH with arterial stiffness as assessed by cfPWV and high ankle brachial index values^18^. Reasons for the discrepancy in results could be the differences of study population. In the above study, only 12% of CKD patients (eGFR <60 mL/min/1.73 m^2^) were included and only 10.5% participants had serum bicarbonate concentration <23mEq/L. Therefore, there may be differences in characteristics of CKD patients who are prone to metabolic acidosis. In addition, their study population was limited to those aged 70–79.

cfPWV is the most validated method of determining PWV, but requires considerable technical expertise^19^. baPWV provides a more convenient and reproducible method of measuring arterial stiffness and uses an oscillometric method to record pulse waves in brachial and posterior tibial arteries^9^, and in the present study, baPWV values were used to assess arterial stiffness. Furthermore, baPWV results have been shown to be well correlated with cfPWV results and to exhibit similar associations with cardiovascular disease risk factors^11,12^. Given the simplicity of the technique, baPWV provides an excellent means of evaluating arterial stiffness in large-scale multicenter population studies.

Metabolic acidosis may be associated with arteriosclerosis and arterial stiffness by several mechanisms. The deleterious effects of systemic acidosis on bone have long been evaluated^20^, and the skeleton is known to play a homeostatic role in terms of buffering acid loads. Previous experimental studies have demonstrated metabolic acidosis induces bone resorption and increases calcium release^21,22^. Since bone acts as a buffer in response to acidosis, metabolic acidosis evokes calcium and phosphate efflux from bone into blood^13,15,23^. However, it is hard to simply explain that calcium and phosphate efflux contribute to vascular calcification, and the link between metabolic acidosis and vascular calcification is complicated. For example, metabolic acidosis increases calcium and phosphate solubilities, decreases parathyroid hormone secretion, inhibits alkaline phosphatase, down-regulates phosphate uptake transporter, and acts to prevent of vascular calcification^24,25^. A previous study showed metabolic alkalosis induced using high bicarbonate dialysate increased the risk of metastatic calcification^26^ and that the absence of metabolic acidosis independently predicted the presence of abdominal aorta calcification in pre-dialysis CKD patients^27^. However, in another study, pre-hemodialysis bicarbonate levels were found to be inversely associated with coronary artery calcification scores^23^. Other adverse effects of metabolic acidosis such as arterial inflammation and insulin resistance could also contribute to vascular calcification. Metabolic acidosis is known to aggravate the development of arterial wall inflammation, which is associated with cytokine release^22,28,29^, and possibly induces arterial calcification. Vascular calcification induces arterial stiffness^17,30,31^. In addition, arterial stiffness may be associated with decreased muscle mass and insulin resistance^7,32^. The adverse effects of metabolic acidosis include muscle protein degradation, muscle wasting, and glucose tolerance impairment, and thus, metabolic acidosis may cause arterial stiffness.

Cardiovascular disease is an important cause of morbidity and mortality in CKD patients^33^. CKD is associated with intimal (atherosclerosis) and medial calcification (arteriosclerosis), though medial calcification is more prevalent^29^. Increased arterial stiffness is associated with higher risks of cardiovascular events^34,35^, mortality^36,37^, and renal progression^38^. Therefore, it is important to prevent arterial stiffness.

Available guidelines provide different target ranges for serum bicarbonate. The National Kidney Foundation–Kidney Disease Outcomes Quality Initiative (NKF-KDOQI) and Care of Australians with Renal Impairment (CARI) guidelines recommend serum bicarbonate be maintained at ≥22 mmol/L^39^, whereas the 2013 KDIGO Guideline recommends maintaining serum bicarbonate within the reference range for the clinical laboratory (23–29 mmol/L)^40^. The Chronic Renal Insufficiency Cohort (CRIC), an observational longitudinal study of CKD patients, reported patients with CKD and a serum bicarbonate concentration >24 mmol/L had a higher prevalence of congestive heart failure than those with a serum concentration of ≤24 mmol/L^41^, and that CKD patients and a serum bicarbonate concentration of >26 mmol/L exhibited increased mortality^42^. These findings indicate overshooting serum bicarbonate targets could adversely affect clinical outcomes. Further studies are needed to determine optimal serum bicarbonate concentrations and whether metabolic acidosis correction to an optimal range improves arterial stiffness.

The inclusion of a large number of pre-dialysis CKD patients is undoubtedly a strength of our study, but it also has several limitations. First, we cannot exclude the possibility of residual confounders because of the observation nature of the study. Second, its cross-sectional design prevented our exploring the causal relationship and the mechanistic links between metabolic acidosis and arterial stiffness. Third, a single serum bicarbonate measurement was used to predict the presence of arterial stiffness. In a recent pilot randomized study, treatment with oral sodium bicarbonate significantly improved vascular endothelial dysfunction with CKD stage 3b and 4^43^. However, our study is observational cohort study. In addition, we did not investigate the effects of alkali therapy (e.g., sodium bicarbonate, sodium citrate, sodium lactate, calcium citrate, *etc*.) on the incidence of metabolic acidosis or on baPWV values. No patient included was taking non-calcium based phosphate binders (e.g., sevelamer hydrochloride or sevelamer carbonate), which affect acid-base balance, and the possibility of dilutional acidosis in metabolic acidosis was excluded because we did not enroll acutely ill patients requiring large volume resuscitation.

In conclusion, metabolic acidosis, as defined by low serum bicarbonate value, was found to be associated with high baPWV in this cohort of Korean pre-dialysis, CKD patients. Further studies are warranted to elucidate the nature of the causal relationship between serum bicarbonate and arterial stiffness and to determine whether correction of acidosis improves arterial stiffness.

## Methods

### Study design and population

The KoreaN Cohort Study for Outcome in Patients With Chronic Kidney Disease (KNOW-CKD) was a Korean multicenter prospective cohort study that enrolled subjects with CKD from stage 1 to 5 (predialysis) from nine clinical centers of major university-affiliated hospitals in Korea. Details about the study design and methods are described elsewhere^44^. Of the 2,238 participants enrolled in the KNOW-CKD study from June 2011 to January 2016, we included 1,659 subjects with serum bicarbonate level and baPWV test results obtained at enrollment. To determine if there were any important selection bias, we compared the clinical characteristics of subjects excluded from the study and those included in the study. The study protocol was approved in 2011 by the ethical committee of each participating clinical center, that is, the Institutional Review Boards of Seoul National University Hospital (1104-089-359), Seoul National University Bundang Hospital (B-1106/129-008), Yonsei University Severance Hospital (4-2011-0163), Kangbuk Samsung Medical Center (2011-01-076), Seoul St. Mary’s Hospital (KC11OIMI0441), Gil Hospital (GIRBA2553), Eulji General Hospital (201105-01), Chonnam National University Hospital (CNUH-2011-092), and Pusan Paik Hospital (11–091). In addition, the study was conducted in accordance with the principles of the Declaration of Helsinki and all study subjects provided written informed consent.

### Clinical data collection and laboratory measurements

Baseline clinical characteristics such as age, gender, body mass index (BMI), cause of CKD, smoking, comorbidities, and laboratory values at enrollment were extracted from an electronic data management system (http://www.phactaX.org) with assistance from the Division of Data Management at Seoul National University Medical Research Collaborating Center. HTN was defined as a systolic blood pressure of ≥140 mmHg, a diastolic blood pressure of ≥90 mmHg, or a history of hypertension. DM was defined as a fasting serum glucose of ≥126 mg/dL or a history of DM or anti-diabetic treatment. Cardiovascular disease was defined as any history of coronary artery disease, cerebrovascular disease, congestive heart failure, arrhythmia, or peripheral vascular disease. The following laboratory variables were measured using a ≥8-hour fasting blood sample at each participating laboratory; TCO_2,_ hemoglobin, uric acid, albumin, total cholesterol, C-reactive protein, phosphorous, calcium, and intact parathyroid hormone. Serum TCO_2_ was considered a surrogate of serum bicarbonate level^45–47^. Serum creatinine was measured using an isotope dilution mass spectrometry (IDMS)-traceable method^48^ at a central laboratory (Lab Genomics, Seoul, Republic of Korea). eGFR was calculated using the Chronic Kidney Disease Epidemiology Collaboration (CKD-EPI) creatinine equation^49^. Stage 1 to 5 CKD were defined according to KDIGO guidelines^50^. Second voided urine samples were immediately sent to a central laboratory to determine urine creatinine and protein. Urinary protein excretion was quantified using urinary protein-to-creatinine ratio (UPCR, g/g). Estimated dietary protein intake was estimated using the Maroni-Mitch formula: 6.25 × [urine urea nitrogen (g/day) + 0.03 × body weight (kg)] + proteinuria (g/day)^51^, and DPI was calculated by dividing estimated dietary protein intake by body weight (g/kg/day).

### Arterial stiffness

baPWV was used as an arterial stiffness marker^52^, because of ease of measurement, validity, and reproducibility, as previously described in previous studies^9^. baPWV was measured using an automatic wave form analyzer (VP-1000, Colin Co., Komaki, Japan). The subjects were examined after a 5 minute rest in the supine position in a quiet environment with electrocardiogram electrodes placed on both wrists, a microphone placed on the left edge of the sternum to detect heart sounds, and cuffs on brachia and ankles. Bilateral brachial and posterior-tibial arterial pressure waveforms were stored for 10 s by extremities cuffs connected to a plethysmographic sensor and an oscillometric pressure sensor wrapped on both arms and ankles. Electrocardiograms were obtained from electrodes on both wrists. Heart sounds S1 and S2 were checked using a microphone positioned on the left edge of the sternum at the third intercostal space. Time interval between the wave front of the brachial waveform and that of the ankle waveform was defined as the time interval between the brachium and ankle. Distances between baPWV sampling points were calculated automatically and adjusted for patient height. Path lengths from suprasternal notches to brachium and from suprasternal notches to ankle were calculated. Finally, baPWV was defined as: the distance between brachium and ankle divided by the time interval between brachium and ankle. Left and right baPWV values were determined and averaged for the analysis.

### Statistical analyses

Categorical variables were analyzed using the Chi-square test or Fisher’s exact test, and results are presented as frequencies and percentages. Continuous variables were analyzed by one-way analysis of variance (ANOVA) or the Kruskal-Wallis test. The Kolmogorov-Smirnov test was used to determine the normality of distributions. Results are presented as mean ± standard deviation (SD) for normally distributed variables and as median (interquartile range) for variables with a skewed distribution. Log transformation was used to normalize proteinuria and iPTH values. Participants were allocated to four groups by serum bicarbonate level; low, lower normal, higher normal, and high TCO_2_ values were defined as <22, 22–26, 26.1–29.9, and ≥30 mmol/L, respectively; based on considerations of guidelines for CKD management and previous reports^42,53,54^ and normal TCO_2_ range for the clinical laboratory. Metabolic acidosis was defined as a TCO_2_ value of <22 mmol/L. Serum AG was defined as the concentration of sodium minus the sum of chloride and bicarbonate concentrations. A high AG was defined as AG >12 mmol/L. Multivariable linear regression model analysis with adjustment (the enter method) that included variables significant by univariate analysis and other clinically relevant variables was used to investigate the association between serum bicarbonate (continuous or categorical values) and baPWV. *P*-values of < 0.05 were considered statistically significant. SPSS statistical software (SPSS version 20.0, IBM Co., Armonk, NY, USA) was used for descriptive and outcome analyses.

## Supplementary information

Supplemental Table 1

## Data Availability

All data generated or analyzed during this study are included in this published article.
