# Supplementary material for: Metabolic acidosis is associated with pulse wave velocity in chronic kidney disease: Results from the KNOW-CKD Study
Source: Sci Rep. 2019 Nov 6;9:16139. doi: 10.1038/s41598-019-52499-6 (PMC6834555; doi:10.1038/s41598-019-52499-6)
Supplement: Supplementary file 1 — Supplemental Table 1 [file 41598_2019_52499_MOESM1_ESM.docx]

**Metabolic acidosis is associated with pulse wave velocity in chronic kidney disease: Results from the KNOW-CKD Study**

Hyo Jin Kim^1^, Eunjeong Kang^2^, Hyunjin Ryu^2^, Miyeun Han^1^, Kyu-Beck Lee^4^, Yong-Soo Kim^5^, Suah Sung^6^, Curie Ahn^2,3^, Kook-Hwan Oh^2,3*^

^1^Department of Internal Medicine, Pusan National University Hospital, Busan, Korea

^2^Department of Internal Medicine, Seoul National University Hospital, Seoul, Korea

^3^Department of Internal Medicine, Seoul National University College of Medicine, Seoul, Korea

^4^Department of Internal Medicine, Kangbuk Samsung Hospital, Sungkyunkwan University School of Medicine, Seoul, Korea

^5^Department of Internal Medicine, Seoul St. Mary’s Hospital, College of Medicine, The Catholic University of Korea, Seoul, Korea,

^6^Department of Internal Medicine, Eulji Medical Center, Eulji University, Seoul, Korea

**Supplemental Table 1. Clinical characteristics of the subjects excluded from the study and those included in the study**

| Characteristics | Subjects excluded  from this study  (n = 579) | Subjects included  In this study  (n = 1659) | | *P*-value | |
| --- | --- | --- | --- | --- | --- |
| Age (mean ± SD) | 54.5 ± 11.8 | 53.4 ± 12.4 | | 0.050 | |
| Sex, male, n (%) | 347 (59.9) | 1,020 (61.5) | | 0.510 | |
| BMI (kg/m^2^) | 24.5 ± 3.2 | 24.6 ± 3.5 | | 0.575 | |
| SBP (mmHg) | 128.6 ± 18.6 | 127.5 ± 15.3 | | 0.185 | |
| DBP (mmHg) | 77.0 ± 11.7 | | 77.0 ± 10.9 | | 0.525 |
| MAP (mmHg) | 94.2 ± 13.0 | 93.8 ± 11.2 | | 0.910 | |
| Heart rate (/min) | 72.0 ± 12.3 | 73.4 ± 12.9 | | 0.023 | |
| DM, n (%) | 180 (31.1) | 574 (34.7) | | 0.115 | |
| HTN, n (%) | 564 (97.4) | 1586 (95.7) | | 0.060 | |
| Preexisting CV disease, n (%) | 69 (11.9) | 279 (16.8) | | 0.005 | |
| Cause of CKD |  |  | | < 0.001 | |
| DN, n (%) | 53 (9.2) | 387 (23.3) | |  | |
| Hypertension, n (%) | 121 (20.9) | 288 (17.4) | |  | |
| GN, n (%) | 245 (42.3) | 565 (34.1) | |  | |
| PKD, n (%) | 53 (9.2) | 311 (18.7) | |  | |
| Others, n (%) | 29 (5.0) | 108 (6.5) | |  | |
| eGFR(mL/min/1.73m^2^) | 52.4 ± 29.6 | 53.3 ± 31.1 | | 0.529 | |
| Hemoglobin (g/dL) | 12.8 ± 2.0 | 12.8 ± 2.0 | | 0.856 | |
| Uric acid (mg/dL) | 7.0 ± 1.8 | 7.0 ± 1.9 | | 0.783 | |
| Albumin (g/dL) | 4.1 ± 0.4 | 4.2 ± 0.4 | | 0.001 | |
| Total cholesterol (mg/dL) | 172.8 ± 40.4 | 174.6 ± 38.8 | | 0.366 | |
| CRP, median, (Q1, Q3) (mg/L) | 0.6 (0.6, 1.6) | 0.6 (0.2, 1.7) | | <0.001 | |
| Phosphorus (mg/dL) | 3.7 ± 0.7 | 3.7 ± 0.7 | | 0.166 | |
| ^*^Corrected Ca (mg/dL) | 8.9 ± 0.5 | 9.0 ± 0.4 | | < 0.001 | |
| iPTH, median (Q1, Q3) (pg/mL) | 50.9 (33.7, 83.7) | 56.2 (36.9, 94.7) | | 0.657 | |
| UPCR (Q1, Q3) (g/g) | 0.49 (0.15, 1.46) | 0.58 (0.19, 1.83) | | 0.987 | |

^*^Corrected Ca (mg/dL) = measured total Ca (mg/dL) + 0.8ⅹ[4 –measured serum albumin (g/dL)]

SD, standard deviation; BMI, body mass index; SBP, systolic blood pressure; DBP, diastolic blood pressure; MAP, mean arterial pressure; DM, diabetes mellitus; HTN, hypertension; CV, cardiovascular; CKD, chronic kidney disease; DN, diabetic nephropathy; PKD, polycystic kidney disease; eGFR, estimated glomerular filtration rate as determined by the CKD-EPI creatinine equation; CRP, C-reactive protein; Ca, calcium; iPTH, intact parathyroid hormone; UPCR, urine protein creatinine ratio
